# Cardiorenal syndrome: Pathophysiology as a key to the therapeutic approach in an under‐diagnosed disease

**DOI:** 10.1002/jcu.23265

**Published:** 2022-10-11

**Authors:** Maria Prastaro, Ermanno Nardi, Stefania Paolillo, Ciro Santoro, Antonio L. M. Parlati, Paola Gargiulo, Christian Basile, Davide Buonocore, Giovanni Esposito, Pasquale Perrone Filardi

**Affiliations:** ^1^ Department of Advanced Biomedical Sciences University of Naples Federico II Naples Italy

**Keywords:** acute kidney disease, cardiorenal syndrome, cardiovascular disease, chronic kidney disease, heart failure

## Abstract

Cardiorenal syndrome is a clinical condition that impacts both the heart and the kidneys. One organ's chronic or acute impairment can lead to the other's chronic or acute dysregulation. The cardiorenal syndrome has been grouped into five subcategories that describe the etiology, pathophysiology, duration, and pattern of cardiac and renal dysfunction. This classification reflects the large spectrum of interrelated dysfunctions and underlines the bidirectional nature of heart‐kidney interactions. However, more evidence is needed to apply these early findings in medical practice. Understanding the relationship between these two organs during each organ's impairment has significant clinical implications that are relevant for therapy in both chronic and acute conditions. The epidemiology, definition, classification, pathophysiology, therapy, and outcome of each form of cardiorenal syndrome are all examined in this review.

## Graphical abstract

Cardiorenal syndrome is a pathophysiological disorder that involves both heart and kidneys, in which acute or chronic dysfunction of one organ may induce acute or chronic dysfunction of the other. In both chronic and acute situations, an appreciation of the interaction between heart and kidney has practical clinical implications. Authors consider useful differentiating for clinical reasons the acute (CRS‐1 and CRS‐3), chronic (CRS‐2 and CRS‐4), and systemic (CRS‐5) settings.

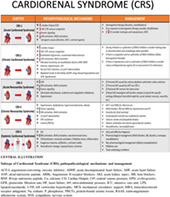

## 1 INTRODUCTION

The term CRS was coined to describe the connection between the cardiovascular (CV) and renal systems, which has long been recognized as interconnected. As early as 1913, Rowntree et al. documented effects of the renal venous congestion on renal activity in dogs.^1^ These observations were corroborated by Winton et al. in 1937, assuming that this partial limitation of urine outflow in the tubules can be the consequence of the increased intrarenal pressure.^2^ In 1949, Blake and colleagues^3^ widely researched the impact of raising renal venous pressure on renal circulation, sodium, and water excretion. In 2004, the National Heart, Lung, and Blood Institute's Working Group attempted to describe CRS as the result of a strong connection between the cardiovascular system and the kidneys that expand circulation volume, worsening heart failure (HF) and renal disease.^4^ In 2008, the Acute Dialysis Quality Initiative (ADQI) proposed a model based on the disease's primum movens, classifying CRS in five subcategories.^5^, ^6^ Renal insufficiency can severely impact on the cardiac function while a damaged heart has several detrimental consequences on the kidney function. In both acute and chronic conditions, knowing the link between the heart and the kidney has clinical implications. In the present review, we analyze the pathophysiology, clinical implications, and treatment of CRS in the acute and chronic settings, pointing the importance of a pathophysiological approach to the disease in order to choose the optimal therapeutic path.

## 2 EPIDEMIOLOGY, DEFINITION, AND CLASSIFICATION

Cardiovascular disease (CVD) is a considerable concern for patients with chronic kidney disease (CKD), accounting for 43.6 percent of all fatalities in end‐stage renal failure (ESRD).^7^ In the United States, one in every three adults has been diagnosed with a CV disease such as hypertension, ischemic heart disease, heart failure, cerebrovascular disease, or congenital heart disease.^8^ Coronary ischemic disease and left ventricular hypertrophy are found in 40% and 75% of ESRD patients, respectively. The incidence of any stage of CKD in the United States has recently been assessed at 13%, which amounts to nearly 30 million patients. CV disease is thought to account for more than half of all fatalities in CRS patients, 10–20 times greater than in age‐matched non‐CRS individuals.^9^, ^10^ The implications of a damaged heart on kidney failure were previously emphasized in suggested definitions of CRS. However, now it is established that both the heart and the kidney might be the first site of injury.^11^ So, CRS can be caused by the simultaneous dysfunction of both the heart and the kidney, independently of which one was damaged first or their previous functional condition. The ADQI working group has proposed a categorization scheme comprising five subtypes to classify this disease in an academic manner.^5^ This five‐point partition is focused on: (a) if the heart, kidneys, or a third separate disease involving both organs is the primary site of damage, (b) whether the cause is chronic or acute. We can define CRS as a cardiac dysfunction leading to kidney function impairment and renocardiac syndrome as a primary renal dysfunction leading to cardiac function impairment.^12^, ^13^ The five CRS subtypes are summarized in Table 1. In 2013, Hatamizadeh et al. proposed an alternative system mostly based on pathophysiological processes such as haemodynamic, neurohumoral, and iron and mineral metabolism‐related mechanisms.^14^ However, it is more hard to apply this classification in clinical practice compared to the ADQI working group classification, and for this reason it is now less popular. When discussing modification in kidney function in HF, the terms “worsening of renal function” (WRF) and “acute kidney injury” (AKI) are frequently employed. The criteria of WRF and AKI based on biomarker alterations are summarized in Table 2.

**TABLE 1:** Subtypes of cardiorenal syndrome (CRS) according to acute dialysis quality initiative working group classification

Cardiorenal types	Characteristics	Causes of morbidity
Type 1 (acute cardiorenal)	Acute worsening of heart function leading to acute kidney injury and/or dysfunction	Cardiogenic shock and AKI, acute decompensated heart failure (ADHF) resulting in AKI
Type 2 (chronic cardiorenal)	Chronic abnormalities in heart function leading to progressive kidney injury and/or dysfunction	Chronic heart failure with left ventricular remodeling and dysfunction leading to CKD
Type 3 (acute renocardiac)	Acute worsening of kidney function leading to acute heart injury and/or dysfunction	Heart failure in the setting of AKI from volume overload, inflammatory surge (es: pericarditis) and accompanying metabolic disturbances
Type 4 (chronic renocardiac)	Chronic kidney disease leading to heart injury, disease, and/or dysfunction	Myocardial remodeling and heart failure from CKD‐associated cardiomyopathy
Type 5 (secondary cardiorenal)	Systemic conditions leading to simultaneous injury and/or dysfunction of heart and kidney	Diabetes, amyloidosis, sepsis, cirrhosis, hypertension, vasculitis

Abbreviations: ADHF, acute decompensated heart failure; AKI, acute kidney injury; CKD, chronic kidney disease.

**TABLE 2:** Definitions of WRF and AKI

	Definition of WRF	
GFR based definitions	Cystatin‐C based definitions	Creatinine based definitions
≥25% decrease >5 ml/min/1.73mq per year decrease	>0.3 mg/dl increase	>0.3 mg/dl increase and >25% increase >0.5 mg/dl increase 1.5x baseline >25% increase + above 2.0 mg/dl

	Definition of AKI (KDIGO)	
Stages	UO component	SCr component
Stage 1	<0.5 ml/kg/h for 6–12 h	SCr 1.5–1.9x baseline over 7 days or absolute increase ≥0.3 mg/dl over 48 h
Stage 2	<0.5 ml/kg/h for ≥12 h	SCr 2.0–2.9x baseline
Stage 3	<0.3 ml/kg/h for ≥24 h or anuria for ≥12 h	SCr 3.0x baseline or increase above ≥4.0 mg/dl or RRT

*Note*: To calculate serum creatinine in mg/dl to μmol/L multiply with 88.4. For AKI criteria, if urine output and serum creatinine stage do not correspond to the same stage, patients are classified in the worse stage. AKI criteria have a stage 1, stage 2, and stage 3.

Abbreviations: AKI, acute kidney injury; GFR, glomerular filtration rate; KDIGO, kidney disease: improving global outcomes; RRT, renal replacement therapy; SCr, serum creatinine; UO, urine output; WRF, worsening of renal function.

## 3 PATHOPHYSIOLOGY

The various pathophysiological processes involved in such a complicated disease contribute to the challenges in describing, investigating, and treating CRS. Table 3 lists and describes all of the different pathophysiological processes of CRS. It is useful to distinguish between acute, chronic, and systemic settings for therapeutic reasons. Therefore, we will first discuss about CRS in the acute setting, then CRS in the chronic setting (CRS‐2 and CRS‐4), and finally approach to systemic CRS (CRS‐5).

**TABLE 3:** Pathophysiological factors of CRS

Mechanisms	Mediators	Heart effects	Renal effects
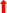 Central venous and intra‐abdominal pressures	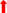 Water and sodium retention 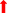 Activation of RAAS/SNS	Acute/chronic HF Remodeling heart	Renal venous congestion 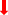 GFR
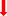 Cardiac output and cardiac index	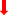 Vascular resistance 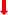 Perfusion pressure Peripheral vasodilatation	RAAS/SNS activation, detrimental to the heart Cardiac ischemia from reduced perfusion	Reduced renal perfusion Renal ischemia
Neurohormonal dysregulation: RAAS activation, SNS activation, AVP	Impaired baroreceptor reflexes 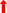 Renin, ang II and aldosterone secretion 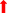 ET‐1 expression Oxidative stress	Ventricular hypertrophy Ventricular disfunction Infiammation with fibrotic effect Hypertension	Arteriolar vasoconstriction 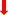 GFR 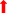 Water and sodium reabsorption Infiammation with fibrotic effect
Oxidative stress	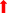 ROS production 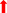 NADPH oxidase activity 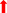 Uremic toxin‐mediated cytokines release	Ventricular hypertrophy Accelerated atherosclerosis Endothelial dysfunction Infiammation and fibrosis	Endothelial dysfunction Accelerated atherosclerosis Fibrosis Infiammation and interstitial fibrosis
Infiammatory mediators	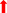 TNF‐alpha, IL‐1 family, IL‐6, PCR	Accelerated atherosclerosis Infiammation and fibrosis Left ventricular dysfunction and hypertrophy Myocardial cells apoptosis	Infiammation and fibrosis Atherosclerosis Glomerular damage by mesangial cells apoptosis
Renal failure related disturbances	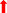 PBUTs 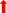 Chronic infiammatory cytokines Oxidative stress FGF23 and Ca‐P abnormalities Anemia (EPO resistance) Acidemia Electrolyte disturbances Coagulation imbalances	Endothelial dysfunction Atherosclerosis and vascular calcification Left ventricular dysfuncion and hypertrophy Ischemia Arrythmias	Atherosclerosis Infiammation 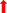 Interstitial and perivascular fibrosis
Iatrogenic factors	Drugs (ACE‐I, ARBs, ARNI, diuretics) Contrast agents	Hypotension	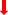 GFR Nephrotoxicity

Abbreviations: ACEi, angiotensin‐converting enzyme inhibitor; ang II, angiotensin II; ARBs, angiotensin receptor blockers; ARNI, angiotensin receptor‐neprilysin inhibitor; FGF‐23, fibroblast growth factor 23; GRF, glomerular filtration rate; HF, heart failure; PBUTs, protein‐bound uremic toxins; PCR, protein C‐reactive; RAAS, renin‐angiotensin‐aldosterone system; ROS, reactive oxygen species; SNS, sympathetic nervous system.

### 3.1 CRS in the acute setting

**FIGURE 1:**
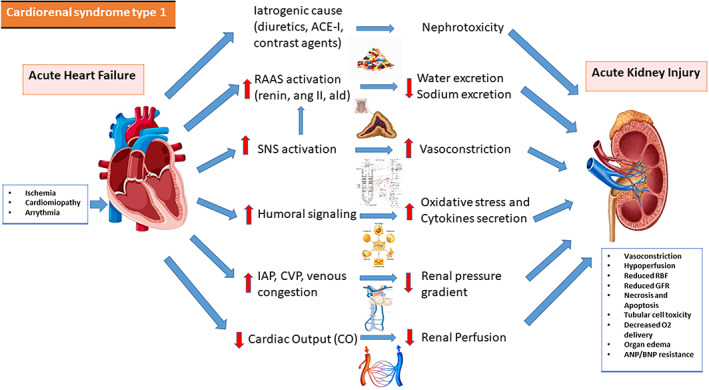
Pathophysiology of CRS‐1. Interaction between heart and kidney in cardiorenal syndrome type 1. ACE‐I, angiotensin‐converting enzyme inhibitor; ald, aldosterone; ang II, angiotensin II; ANP, atrial natriuretic peptide; BNP, B‐type natriuretic peptide; CVP, central venous pressure; GFR, glomerular filtration rate; IAP, intra‐abdominal pressure; KIM‐1, kidney injury molecule‐1; L‐FABP, liver‐type fatty acid‐binding protein; RAAS, renin‐angiotensin‐aldosterone system; RBF, renal blood flow; SNS, sympathetic nervous system

**CRS‐1** is defined by a rapid reduction of the cardiac function, which leads to AKI. AKI affects about a quarter of individuals with acute decompensated heart failure (ADHF). The prevalence of CRS‐1 was 25.4 percent, categorizing renal outcome by AKI; more than 30 percent of ADHF hospital admissions had a story of renal insufficiency, and 20% used to have an excess of serum creatinine levels of 2.0 mg/dl.^15^ WRF has traditionally been linked to kidney hypoperfusion caused by reduced cardiac output (CO). CO's influence in the acute setting is heterogeneous, and it may play a role in some of the most serious types of ADHF, although it is unlikely to play a significant role in the majority of patients. ADHF causes volume overload as well as an increase in central venous pressure (CVP). Increased venous pressures and congestion reduce the blood flow gradient through the glomerular capillary system, resulting in slow intravascular flow, glomerular functional impairment, and decreased urine production. CVP increases have also been demonstrated to be directly linked to renal function.^11^ The renin‐angiotensin‐aldosterone system (RAAS) has a role in renal damage development and worsening HF. This neurohormonal pathways are engaged in HF patients to restore tissue perfusion. A rise in renin levels can lead to a greater production of a rise in angiotensin II (Ang II), which has numerous deleterious systemic consequences on the kidneys. An increase in renal plasma flow filtrated through the glomerulus and vasoconstriction of renal efferent arterioles are promoted by Ang II, resulting in lower hydrostatic pressure, increased salt reabsorption in the proximal tubule and higher peritubular oncotic pressure. Ang II also enhances the production of endothelin‐1 (ET‐1) in the kidney and increases aldosterone‐mediated salt reabsorption in the distal tubules. ET‐1 is a powerful vasoconstrictor, proinflammatory, and profibrotic peptide that causes kidney injury through pathologic modifications. Furthermore, volume expansion, sympathetic nervous system (SNS) and RAAS activation are all involved in the amplification of the oxidative stress. Oxidative stress markers were measured in patients with ADHF who had AKI, and those who had CRS had significantly increased levels of oxidative stress markers.^16^ Furthermore, in ADHF, the introduction and up‐titration of RAAS blockers, as well as intense diuretic therapy, may cause WRF, which usually occurs later in the course of HF hospitalization. During an ADHF hospitalization, nephrotoxic drugs such as iodized contrast, non‐steroidal anti‐inflammatory medicines (NSAIDs) and some antibiotics can also cause WRF. Figure 1 summarizes the pathophysiology of CRS‐1.

**FIGURE 2:**
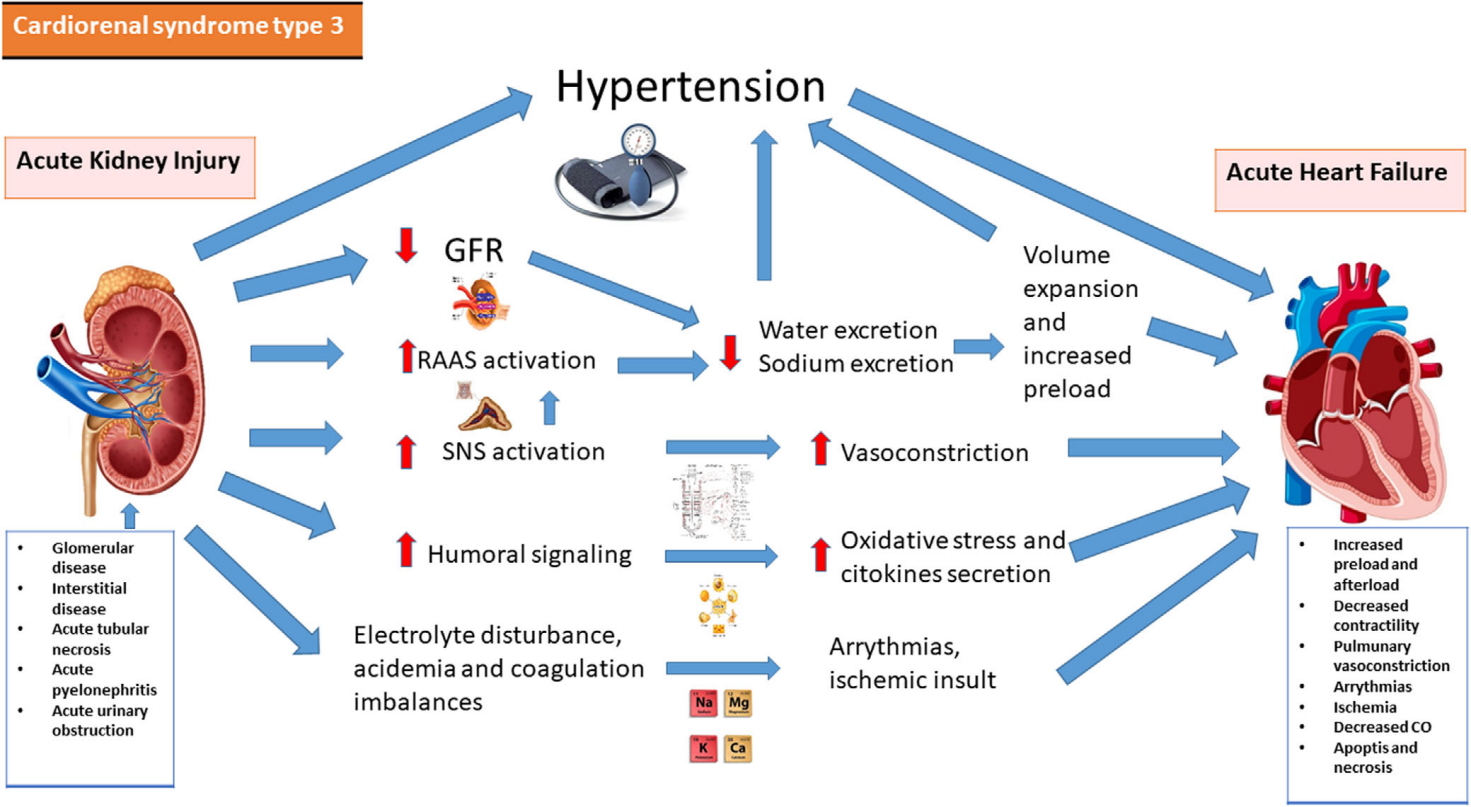
Pathophysiology of CRS‐3. Interaction between heart and kidney in cardiorenal syndrome type 3. CO, cardiac output; GRF, glomerular filtration rate; RAAS, renin‐angiotensin‐aldosterone system; SNS, sympathetic nervous system

**CRS‐3** is identified by a gradual reduction in kidney function as a result of AKI, ischemia, or glomerulonephritis leading acute cardiac impairment. AKI is associated with a 58% increased risk of HF (RR 1.58; 95 percent CI 1.46–1.72), a 40% higher risk of acute coronary syndrome (SCA) (RR 1.40; 95 percent CI 1.23–1.59), and a 15% increased risk of cerebrovascular disease.^17^, ^18^ In AKI patients, RAAS activation results in water and sodium reabsorption, as well as volume expansion and hypertension. Fluid accumulation can culminate in pulmonary edema and a higher risk of death. SNS activation can also causes vasoconstriction. Hyperkalemia can occur as a result of AKI, compromising electrolyte balance and triggering arrhythmias. Increased preload and afterload, decreased contractility, pulmonary vasoconstriction, arrhythmia, and ischemia caused by these variables, in combination with oxidative stress, resulted in ADHF. The pathophysiology of CRS 3 is reported in Figure 2.

### 3.2 CRS in the chronic setting

**FIGURE 3:**
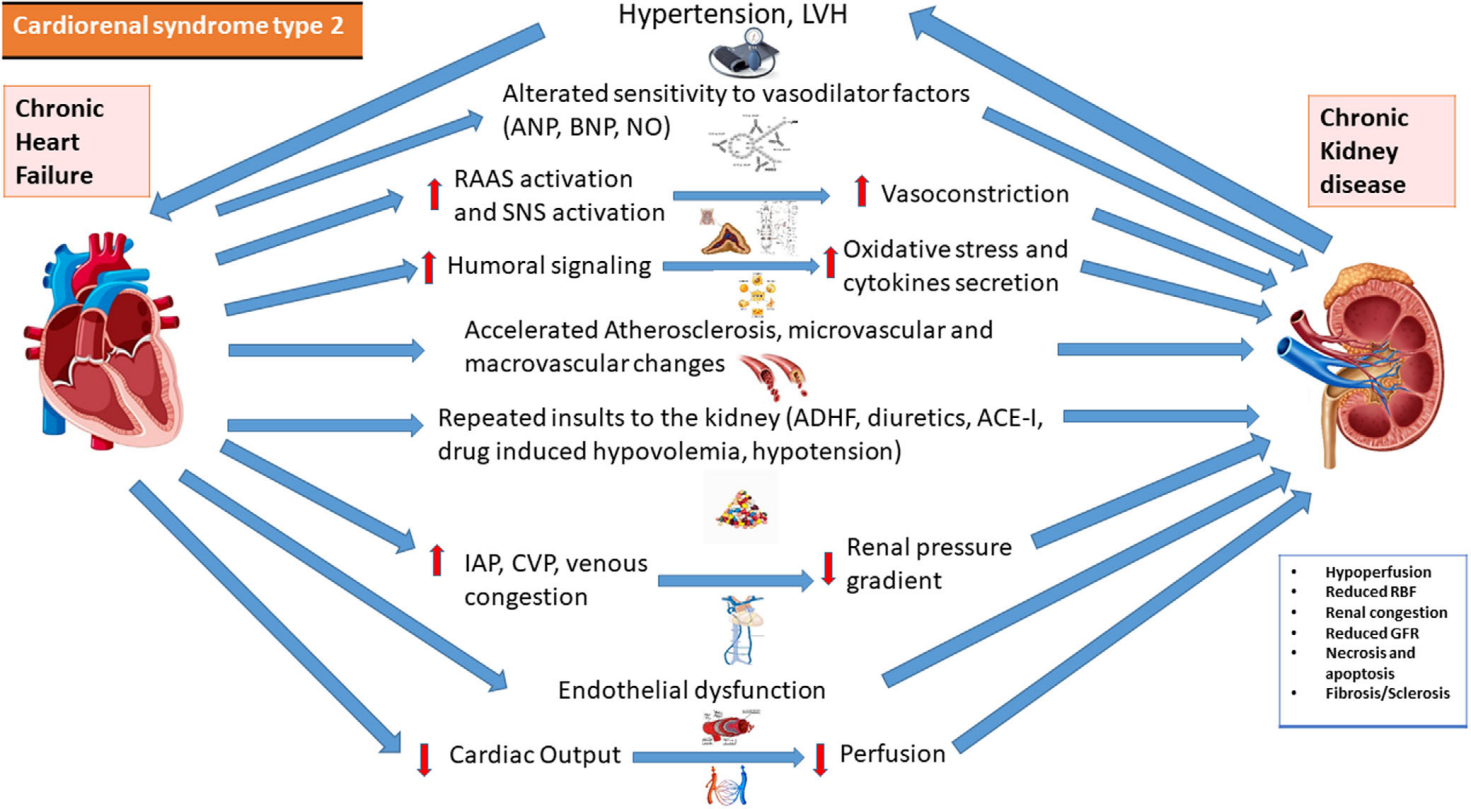
Pathophysiology of CRS‐2. Interaction between heart and kidney in cardiorenal syndrome type 2. ACE‐I, angiotensin‐converting enzyme inhibitor; ADHF, acute decompensated heart failure; ANP, atrial natriuretic peptide; BNP, B‐type natriuretic peptide; CVP, central venous pressure; GFR, glomerular filtration rate; IAP, intra‐abdominal pressure; LVH, left ventricular hypertrophy; RBF, renal blood flow; RAAS, renin‐angiotensin‐aldosterone system; SNS, sympathetic nervous system

**CRS‐2** is identified by a persistent cardiac dysfunction that causes to CKD over time. Both CKD and HF are chronic inflammatory diseases that cause the production of proinflammatory molecules. These biomarkers are important for tissue injury in both systems, which results in fibrosis and cell death. CKD is common in HF, with a prevalence ranging from 20% to 57 percent in chronic HF patients.^18^ HF with preserved and reduced ejection fraction (EF), congenital heart disease, atrial fibrillation (AF) and ischemic heart disease are all underlying diseases in CRS‐2. CKD must appear secondarily to chronic HF to be classified as CRS‐2. Oxidative stress and chronic infiammation have significant weight. TNF‐alpha and IL‐6 increase the production of monocyte chemiotactic factors in the interstitium of the kidneys, promoting the concentration of inflammatory cells in the interstitium. TNF‐alpha also causes mesangial cell death, which damages the glomerulus. C‐reactive protein (CRP), an acute phase protein, demonstrated to have a role in the pathophysiology of atherosclerosis with multiple pathways.^19^ Erythropoietin deficiency is frequently linked to CKD. Palazzuoli et al. observed that erythropoiesis stimulating drugs can improve cardiac function and lower left ventricle size and volume when used to treat HF, CKD, and anemia.^20^ In the RED‐HF study, on the other hand, treating anemia with darbepoetin alfa did not decrease the risk of mortality or hospitalization in patients with systolic HF who were already on medication. Furthermore, people who received darbepoetin alfa had a higher probability of thromboembolism.^21^ Patients with severe anemia (hemoglobin less than 9.0 g/dl) are excluded from this study. As a result, an increase in hemoglobin levels could support Palazzuoli thesis's in case of severe anemia. Figure 3 summarizes the pathophysiology of CRS‐2.

**FIGURE 4:**
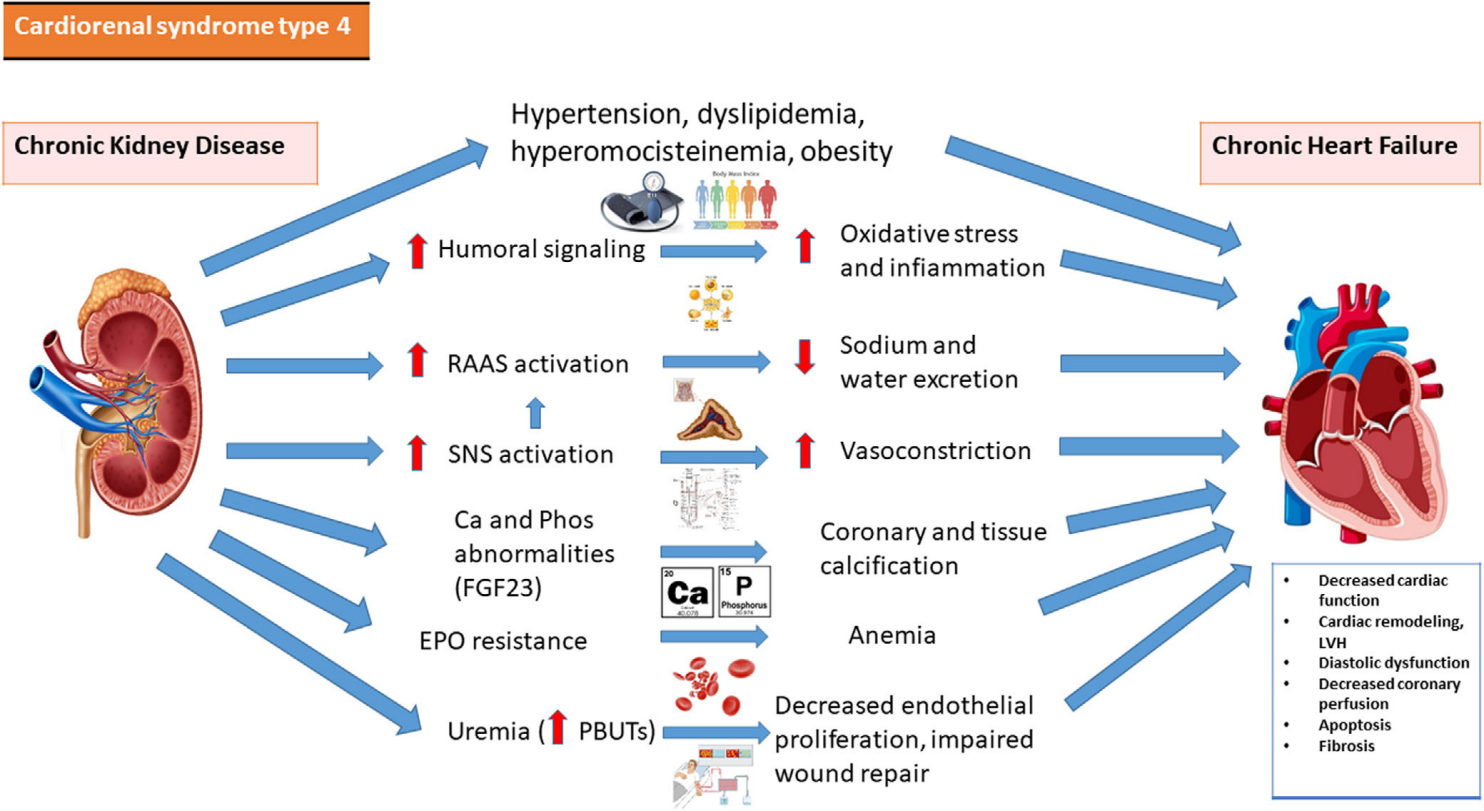
Pathophysiology of CRS‐4. Interaction between heart and kidney in cardiorenal syndrome type 4. Ca, calcium; EPO, erythropoietin; LVH, left ventricular hypertrophy; PBUTs, protein‐bound uremic toxins; Phos, phosphorus; RAAS, renin‐angiotensin‐aldosterone system; SNS, sympathetic nervous system

**CRS‐4** is defined on the basis of CKD as the cause of cardiac dysfunction. Renal dysfunction is an independent risk factor for CV disease. Ischemic coronary disease affects around 40% of ESRD patients, and chronic HF affects nearly 40% of them.^11^ Vasoconstriction, sodium and water reabsorption, and oxidative stress, RAAS and SNS activation can contribute to CRS‐4. they have been associated to renal damage, reduced proliferation of the endothelium, and worse wound healing, contributing to the progressione of CKD.^22^, ^23^ Furthermore, PBUTs increase oxidative stress in kidneys and heart, resulting in cardiorenal fibrosis. Fibroblast growth factor‐23 (FGF23), a hormone that regulates phosphorus and vitamin D metabolism in the kidney, is a powerful indicator of poor CV prognosis in individuals with CKD and ESRD. Elevated FGF‐23 levels are associated to left ventricular hypertrophy and death in those with severe CKD.^24^ We resume the pathophysiology of CRS‐4 in Figure 4.

### 3.3 Systemic CRS

**FIGURE 5:**
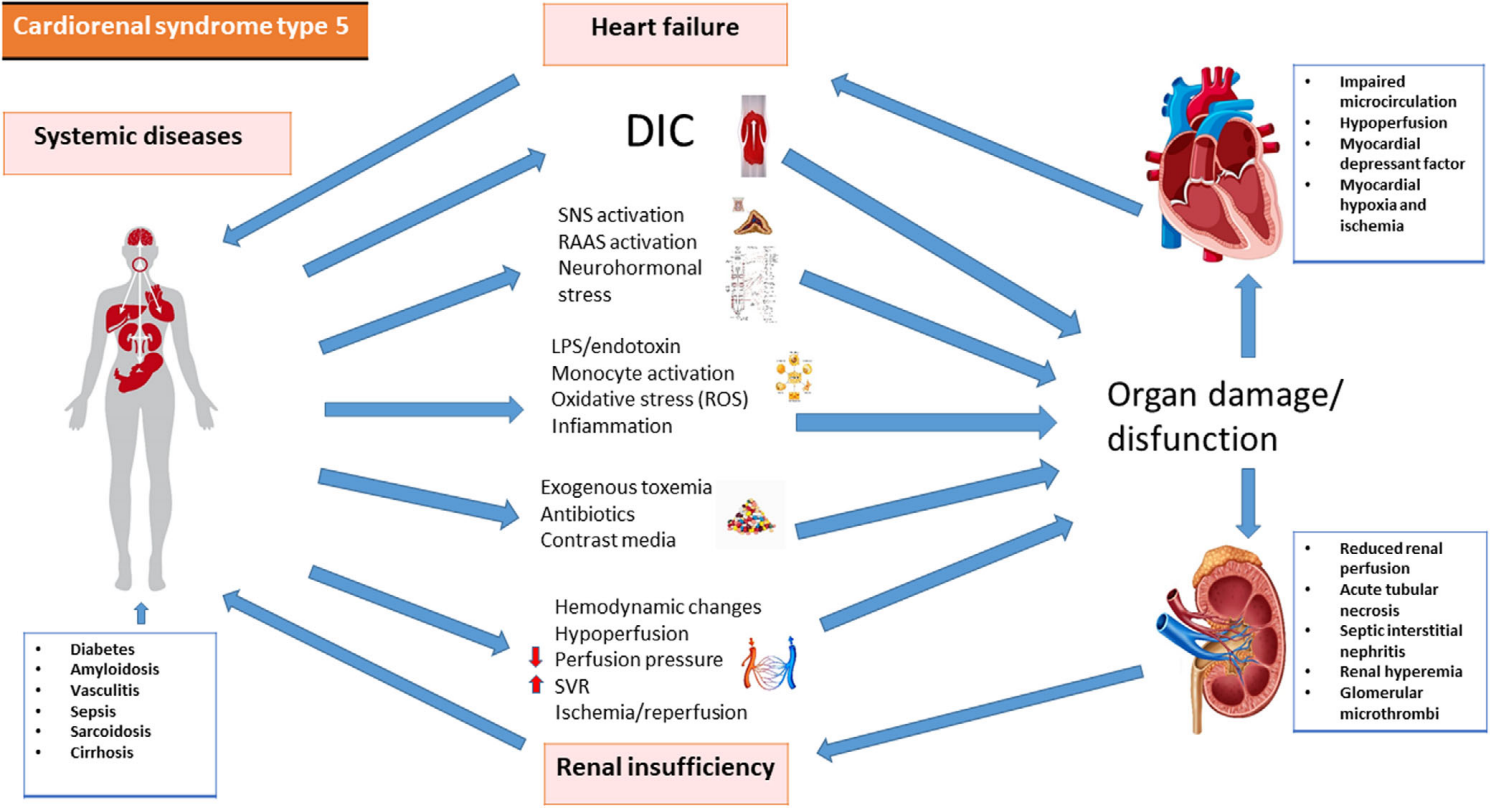
Pathophysiology of CRS‐5. Interaction between systemic disease, heart and kidney in cardiorenal syndrome type 5. DIC, disseminated intravascular coagulation; LPS, lipopolysaccharide; RAAS, renin‐angiotensin‐aldosterone system; ROS, reactive oxygen species; SNS, sympathetic nervous system; SVR, system vascular resistance

**CRS‐5** happens when a systemic illness causes both heart and kidney damage at the same time. Based on the pathophysiological process and severity of the disease, CRS‐5 can be divided into four stages: hyperacute (0–72 h after diagnosis), acute (3–7 days), subacute (7–30 days), and chronic (7–30 days) (beyond 30 days). Systemic disorders that might produce CRS‐5 include sepsis, connective tissue diseases such as lupus, amyloidosis, sarcoidosis, and cirrhosis. According to current understanding, hemodynamic factors and inflammatory markers have a role in the pathophysiology of septic AKI. Renal and cardiac injury are typically mediated by complement factors, inflammatory cytokines and RAAS activation, common destinations for many types of CRS. Organ injury could occur in sepsis due to increased renal vascular resistance, as well as an early rise in oxidative stress and inflammatory cytokines (IL‐6).^25^ Ischemia and inflammatory mediators are the major drivers of AKI in individuals with sepsis: patients with septic shock have higher concentration of prostanoids (like prostacyclin and thromboxane), tumor necrosis factor and IL‐1 with endothelial dysfuncion and lack of autoregulation.^26^ The pathophysiology of CRS‐5 is summarized in Figure 5.

## 4 BIOMARKERS AND DIAGNOSIS

Laboratory and clinical results, ultrasonography, and other radiological investigations are used to diagnose CRS‐1. Biomarkers like Creatinine, for example, are already well‐established role in the diagnosis. New biomarkers including serum and urine neutrophil gelatinase–associated lipocalin (NGAL), kidney injury molecule 1 (KIM‐1), liver‐type fatty acid‐binding protein (L‐FABP), cystatin –C, interleukin 18 (IL‐18) and other renal injury indicators offer new horizons. M arkers of myocardial necrosis, such as troponins I (cTnI) and T (cTnT), and indicators of HF, such as N‐terminal pro B‐type (NT‐proBNP) and the active form B‐type natriuretic peptide (BNP) are frequently utilized in clinical practice. All laboratory biomarkers are reported in Table 4 and briefly subsequently described. In patients with CRS, an ultrasound examination of heart and kidneys could be useful to identity pathologies that require specific treatment like glomerulonephritis or nephrotic syndrome.

**TABLE 4:** Biomarkers useful to support the diagnosis of CRS

Biomarkers of glomerular function	Creatinine Urea Cystatin‐C Albumin Creatinine clearance	Blood and urinary marker Blood marker Blood and urinary marker Urinary marker
Biomarkers of tubular function	KIM‐1 NGAL L‐FABP Electrolytes	Urinary marker Blood and urinary marker Blood and urinary marker Urinary marker
Cardiac biomarkers	cTnT, cTnI NT‐proBNP MR pro‐ANP MR pro‐ADM	Blood markers Blood marker Blood marker Blood marker
Other biomarkers	PRA Aldosterone Il‐1β, IL‐10 ET‐1	Blood marker Blood marker Blood markers Blood marker

Abbreviations: cTnT and cTnI, troponin T and I; ET‐1, endothelin 1; KIM‐1, kidney injury molecule‐1; L‐FABP, liver fatty acid‐binding protein; MR‐proADM, mid‐regional proadrenomedullin; MR‐proANP, Mid‐regional pro‐atrial natriuretic peptide; NGAL, neutrophil gelatinase‐associated lipocalin; NT‐proBNP, N‐terminal pro‐B‐type natriuretic peptide; PRA, plasma renin‐activity.

### 4.1 Biomarkers of glomerular function

Creatinine, which is used to calculate eGFR, and urea plasma levels are the only renal markers with a high recommendation in HF guidelines. Serum urea testing is recommended in all clinical circumstances because it is connected not only to glomerular filtration but also to tubular reabsorption and hence to neurohormonal activity.^27^ The most common endogenous glomerular filtration marker is serum creatinine. Nevertheless, the tubules secrete it in a variety of ways, making it an imprecise indicator of glomerular filtration. To compensate for this flaw, cystatin C was investigated mostly on the assumption that it is filtered by the glomerulus and not secreted by the tubules.^28^ The glomerulus filters serum cystatin‐C, which is then catabolized by renal tubular epithelial cells. Murty and colleagues evaluated the diagnostic value of creatinine and cystatin‐C serum levels in the early stages of AKI.^29^ When compared to the controls, all AKI cases showed higher blood cystatin‐C levels, although half of these had ordinary serum creatinine levels. As a result, cystatin‐C may be a preferable diagnostic marker for renal impairment in the early stages of AKI compared to creatinine. Lately, higher blood cystatin‐C levels have been linked to the development of serious cardiovascular events such as stroke and non‐fatal myocardial infarction in individuals presenting with coronary artery disease.^30^ GFR can be calculated using a multitude of formulae based on serum creatinine, cystatin C, or a mix of the two. In several clinical situations, calculation based purely on serum creatinine is likely sufficient.^31^ Cystatin C, either alone or in conjunction with creatinine, has been demonstrated to increase the link among mortality and CKD risk. Net reclassification improvement (NRI), an indicator that is widely used to assess the prognostic capacity of emerging biomarkers, could be used to quantify the level of improvement in risk stratification for people who are reassigned using cystatin C compared to creatinine.^32^

### 4.2 Biomarkers of tubular function

There is debate on how to quantify tubular function and a wide assortment of biomarkers has been studied. KIM‐1 is a plasmatic and urine indicator for proximal tubule damage, and it is increased following toxic or ischemia renal injury.^33^ When comparing individuals with AKI to individuals without AKI undergoing heart surgery and healthy controls, plasma KIM‐1 concentrations are considerably higher.^34^ 193 individuals with HF or SCA undergoing coronary angiography or other cardiac surgery had comparable findings.^35^ Furthermore, plasma KIM‐1 was increased in HF patients compared to individuals without HF with CKD who received maintenance hemodialysis.^36^ NGAL, commonly referred as lipocalin‐2, is a short peptide secreted from tubular cells as a reaction to acute damage and is the most thoroughly investigated plasma tubular damage biomarker.^37^ Plasma NGAL is linked to infection and inflammation, urine NGAL is assumed to be mainly produced and secreted by tubules. High‐urine NGAL concentrations were linked to the likelihood of CKD advancement and the evolution of ESRD in a large investigation of CKD stages 2–4.^38^ The diagnostic capability of NGAL for renal dysfunction has been also investigated in HF patients.^39^ Palazzuoli et al. compared urinary NGAL in CRS patients and HF patients without renal dysfunction. The levels of urinary NGAL were significantly increased in CRS patients compared to HF patients with no signs of renal abnormalities suggesting that the development of WRF in ADHF patients can be predicted by the elevated urine levels of NGAL and KIM‐1.^40^ Furthermore, in 252 individuals with CKD and without antecedent CV events, NGAL was found to be an independent indicator of CV events such as SCA, aortic dissection, and CV mortality. Liver fatty acid‐binding protein (L‐FABP) has also been proposed as an indicator for tubular function. In ADHF patients developing AKI, urine L‐FABP concentrations were observed to be higher than in ADHF patients not developing AKI.^41^ High urine L‐FABP concentration might be utilized as a predictive indicator for the progression of ERSD and the beginning of CV impairment in CKD, according to a research by Matsui and colleagues.^42^

### 4.3 Urinary biomarkers

Many Indicators of glomerular efficiency (creatinine clearance, urine creatinine), glomerular structure and podocyte function (albuminuria), and urinary indicators of tubular function and damage are tested in the urine (urinary tubular impairment markers, urinary sediment, and electrolytes). Urinary electrolyte levels and urinary volume, particularly, could be used as a functional evaluation to measure tubular function, which could be particularly useful in HF. An early decrease of natriuretic response is related to HF, which leads to the progression of congestion.^30^ Aside from the possible issue of partial collections, we must note that under non‐steady state situations, plasma creatinine levels shift slowly, resulting in mistakes in GFR calculations. As a result, 24‐hour urine collection‐based GFR measurements in chronic HF with stabilized renal function are good approximations of true GFR. Creatinine clearance might be used if GFR prediction based on calculations are uncertain. Albuminuria is a useful method for assessing glomerular integrity. Urinary tubular indicators, in addition to those evaluating glomerular activity and integrity, are frequently employed to assess the development of AKI. Moreover, higher urinary tubular damage indicators fail to detect HF patients with a worse prognosis or reduced diuretic response.^43^ As a result, the use of these urinary tubular damage indicators in patients with HF is restricted.

### 4.4 Cardiac biomarkers

Lately, Osmar and collegues has investigated the predictive efficacy of cTnT in individuals who developed AKI following heart surgery. TnT concentrations were are considerably greater in heart surgery‐induced AKI subjects (*n* = 100) compared to non‐AKI subjects (*n* = 259).^44^ Elevated concentrations of natriuretic peptides (NP) could be induced by renal impairment, but they are well‐established indicators in the diagnosis and prognosis of HF. NP have the capacity to be a relevant diagnostic and prognostic resource in a wide spectrum of CRS types^45^, ^46^: elevated not just in CRS types 1 and 2, but also in CRS‐4, where BNP is a relevant parameter for recognizing acute HF in subjects with CKD and expects to be able to forecast cardiovascular events. In a group of 908 subjects with ADHF, elevated BNP concentrations were linked to the probability of renal impairment.^47^ Elevated plasma concentration of MR pro‐ANP (Mid‐regional pro‐atrial natriuretic peptide) and MR pro‐ADM (mid‐regional proadrenomedullin) were linked to CV events (*n* = 85) including SCA, aortocoronary bypass, cerebrovascular events, and all‐cause mortality in dialysis, according to Gouya and collegues.^48^ Copeptin, the C‐terminal component of arginine vasopressin (AVP), is a plasma protein that is easily detectable. As a result, copeptin became an alternative indicator for AVP in blood, indicating vasopressin activity. In a trial of hemodialysis subjects, the highest percentile of copeptin was linked to a 3.5‐fold increased risk of stroke, a 73% increased risk of sudden death, and a 48% increased risk of all‐cause mortality.^49^ Soluble ST2 (sST2) and Galectin‐3 (a‐galactoside‐binding lectin), both members of the IL‐1 receptor class, are indicators of cardiac stress, remodeling, and fibrosis, and are still being discussed in the field of CRS.^50^

### 4.5 Other biomarkers in CRS patients

Two blood indicators, aldosterone, and plasma renin‐activity (PRA), were used to investigate RAAS activity. Elevated concentrations of aldosterone and PRA shown to be strongly linked with WRF in ADHF.^51^ Ortega‐Hernandez and collegues investigated the relationship between numerous inflammatory markers and renal impairment in subjects who had suffered myocardial damage as a result of a SCA. Higher levels of TnI, interleukins (IL‐1, IL‐10), and ET‐1 are related with WRF in SCA subjects developing AKI, and IL‐6 and ET‐1 seem to have a major role in the interaction among “de novo cardiac and renal impairment.”^52^

## 5 MANAGEMENT AND THERAPEUTIC APPROACH

### 5.1 CRS in the acute setting

**FIGURE 6:**
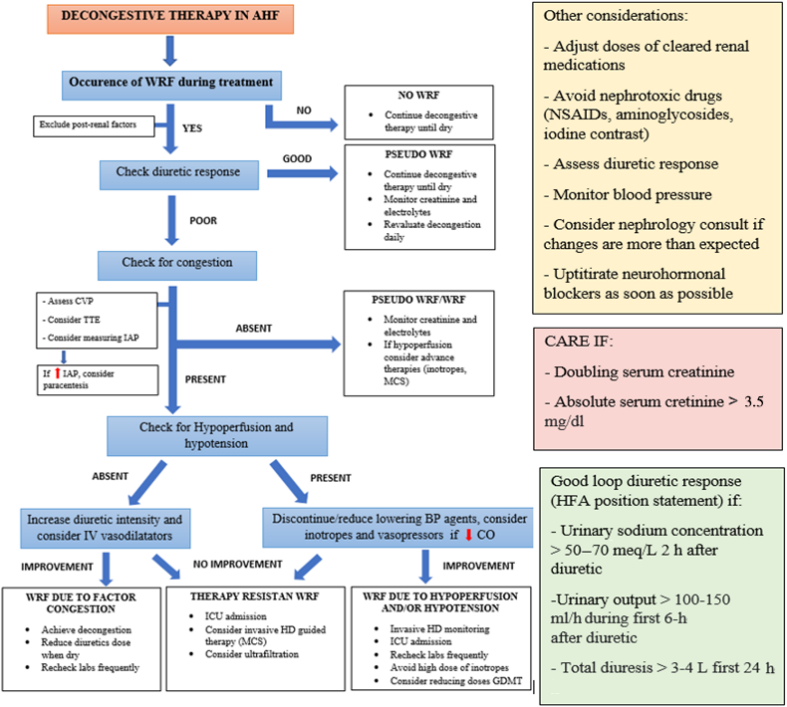
Therapeutic management of CRS‐1. BP, blood pressure; CO, cardiac output; CVP, central venous pressure; GDMT, guideline‐directed medical therapy; HD, hemodynamic; HF, heart failure; IAP, intra‐abdominal pressure; ICU, intensive care unit; IV, intravenous; MCS, mechanical circulatory support; NSAIDS, non‐steroidal anti‐inflammatory drugs; TTE, transthoracic echocardiography; WRF, worsening of renal function

**CRS‐1.** As shown in Figure 6, the Cardio‐Renal Dysfunction Study Group recommends a (frequent) complete examination of kidney function during ADHF with CRS‐1. WRF might be investigated for its onset and possible causes, as well as its link to diuretic response and functional status. In individuals who have a positive diuretic response, every attempt should be addressed to achieve full decongestion, since remnant congestion at discharge is the most common indicator of readmission.^53^, ^54^ Appropriate dosage and early examination of diuretic response by sodium excretion and urine volume estimation, as well as early up‐titration of diuretic dosage, if necessary, are critical.^55^ In addition, in all HF subjects with reduced EF, (re)initiation and up‐titration of RAAS blockers must be explored when feasible. Alternative explanations should be addressed if diuretic response is weak and/or functional status starts to deteriorate for WRF. Initially, reversible conditions such as genitourinary blockage or ascites‐related elevated intra‐abdominal pressure must be ruled out. In hemodynamically stable individuals with ADHF (systolic blood pressure >90 mmHg), the 2021 European Society of Cardiology (ESC) HF guidelines recommend the use of vasodilators. Ultrafiltration should be used as a last resource in individuals with progressive fluid overload and AKI. Utilization of vasopressors, inotropes or interim mechanical assistance must be evaluated in individuals with signs and symptoms of hypotension and hypoperfusion with inadequate diuretic response in ADHF.^26^, ^43^

**FIGURE 7:**
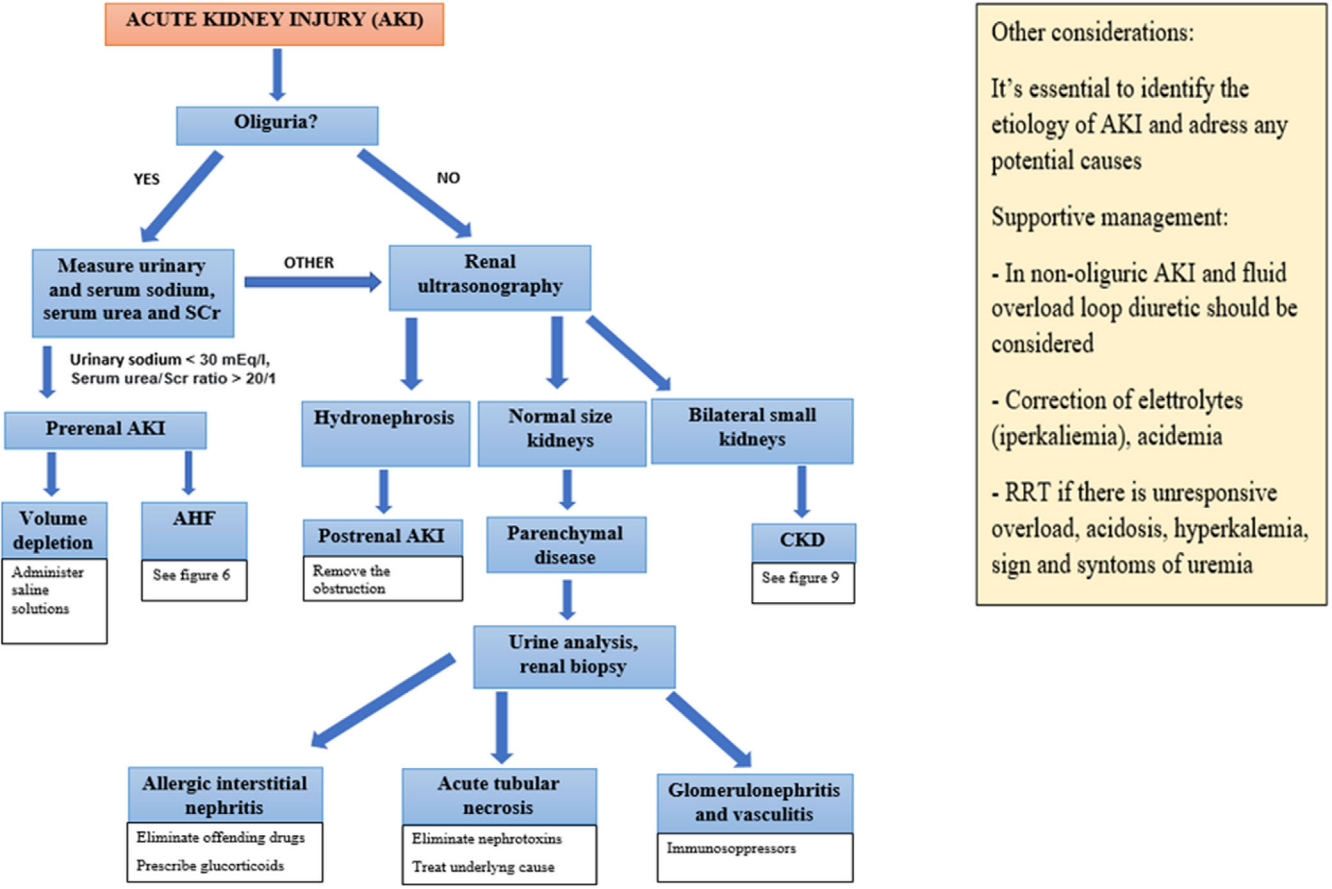
Therapeutic management of CRS‐3. AHF, acute heart failure; AKI, acute kidney injury; CKD, chronic kidney disease; RRT, renal replacement therapy

**CRS‐3.** The cause and severity of AKI, and even the kind of acute cardiac damage, determine the effectiveness of CRS‐3 therapy. The genesis of AKI must be determined, as well as any potentially reversible factors. Obstructive uropathy with surgery, prerenal cause with fluid resuscitation, or acute glomerulonephritis with immunotherapy are all treatable forms of AKI. Administration of loop diuretics is a cornerstone of treatment in the case of non‐oliguric AKI and volume overload. Renal replacement therapy (RRT) may be needed if there is no rapidly recoverable cause and the AKI is significant with consequences such as hyperkalemia, severe acidemia and fluid overload. Observational data supports a link among negative daily fluid balance with RRT and better prognosis in subjects with oliguric AKI with critic fluid overload necessitating RRT.^56^ On the cellular level loop diuretics influence the functioning of the sodium‐potassium‐chloride cotransport system. Because of the incapacity to reabsorb sodium in the loop area of the nephron, the high medullary osmolality is lost, and the kidney's capacity to reabsorb water is reduced. Renal vascular resistance is reduced, resulting in more blood flow to the kidneys. The GFR may have a reversable drop that was due to changes in kidney hydrodynamics. A brief decline in GFR is caused by fluid retention within the nephron lumen caused by higher flow, filtration decrease, and higher intracapsular hydrostatic pressure.^57^ The management of CRS‐3 is summarized in Figure 7.

### 5.2 CRS in the chronic setting

**FIGURE 8:**
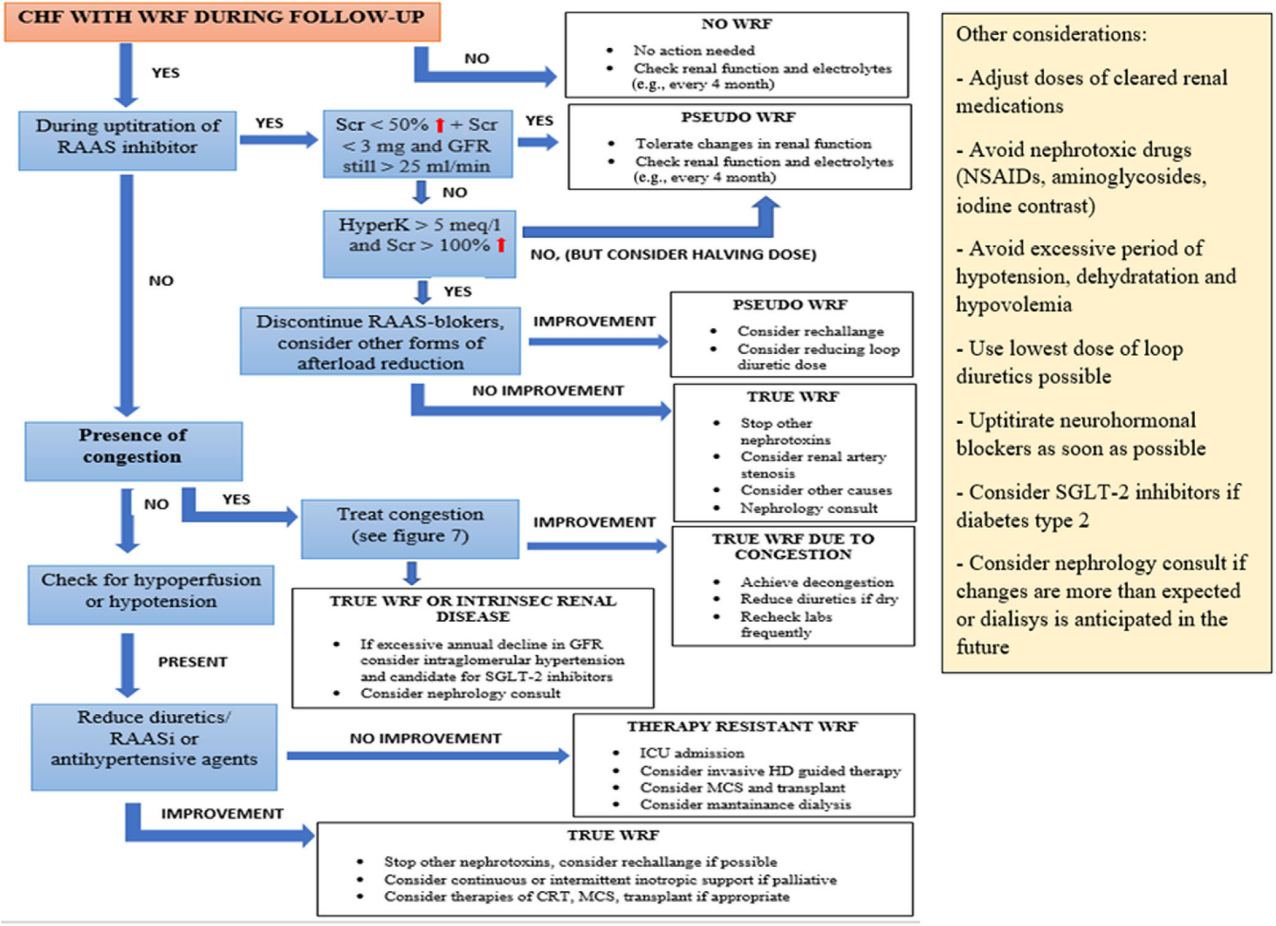
Therapeutic management of CRS‐2. CRT, cardiac resynchronization therapy; FU, follow‐up; GFR, glomerular filtration rate; HD, hemodynamic; ICU, intensive care unit; MCS, mechanical circulatory support; NSAIDS, non‐steroidal anti‐inflammatory drugs; RAAS, renin–angiotensin–aldosterone system; RAASi, renin–angiotensin–aldosterone system inhibitor; Scr, serum creatinine; SGLT2, sodium–glucose co‐transporter 2

**CRS‐2.** Angiotensin‐converting enzyme inhibitors (ACEIs), angiotensin receptor blockers (ARBs), sacubitril/valsartan (ARNI), mineralocorticoid antagonists, and SGLT2 inhibitors were all studied for their involvement in CRS‐2 therapy. After starting RAAS blockers, individuals with baseline CKD see a decrease in their GFR. In most subjects, this decline occurs early, and serum creatinine recovers within 30 percent of initial levels in the large proportion of cases.^58^ ARBs have fewer data, but regardless of the existence of CKD, studies show a comparable effect on outcome.^59^ In individuals with diabetes CKD, ACE‐I, and ARBs proved to be even renoprotective. Sacubitril/valsartan, on the other hand, has been shown to minimize the GFR reduction when compared to enalapril.^60^ Sacubitril/valsartan's favorable effects on death rates are also preserved in subjects with severe CKD. In the same way as ACE‐I and ARBs generate an immediate decline in GFR, the EMPHASIS‐HF study found that MRA introduction provokes an acute decrease in GFR that lasts all the MRA therapy.^61^ Also SGLT‐2 inhibitors induced an initial decrease in eGFR (measured at week 4 of treatment) compared to placebo which showed no significant decrease. During the long‐term follow‐up eGFR remained stable in SGLT2 inhibitor‐treated patients while placebo treatment was accompanied with a progressive decrease in eGFR.^62^, ^63^ It was shown that SGLT2 transporters colocalize and interact with sodium‐hydrogen exchanger 3 (NHE3), which has a prominent part in proximal tubular salt reabsorption. The increased sodium concentration in the loop of Henle following the block of the SGLT2 cotransporters is perceived by the macula densa as a situation similar to that present in hyperfiltration in the initial phases of diabetic nephropathy. Consequently, there is a constriction of the afferent arteriole with reduction of filtrate, filtration rate and glomerular pressure Although a slight dip in eGFR has been reported at the start of SGLT2 inhibitor at the beginning of therapy, eGFR should stabilize over time. This transitory GFR reduction was also recorded in people without CKD who are using SGLT2‐I, and it usually recovers to initial values within months or when the drug is stopped.^64^ Figure 8 shows a strategy to treat CRS‐2 in chronic HF.

**FIGURE 9:**
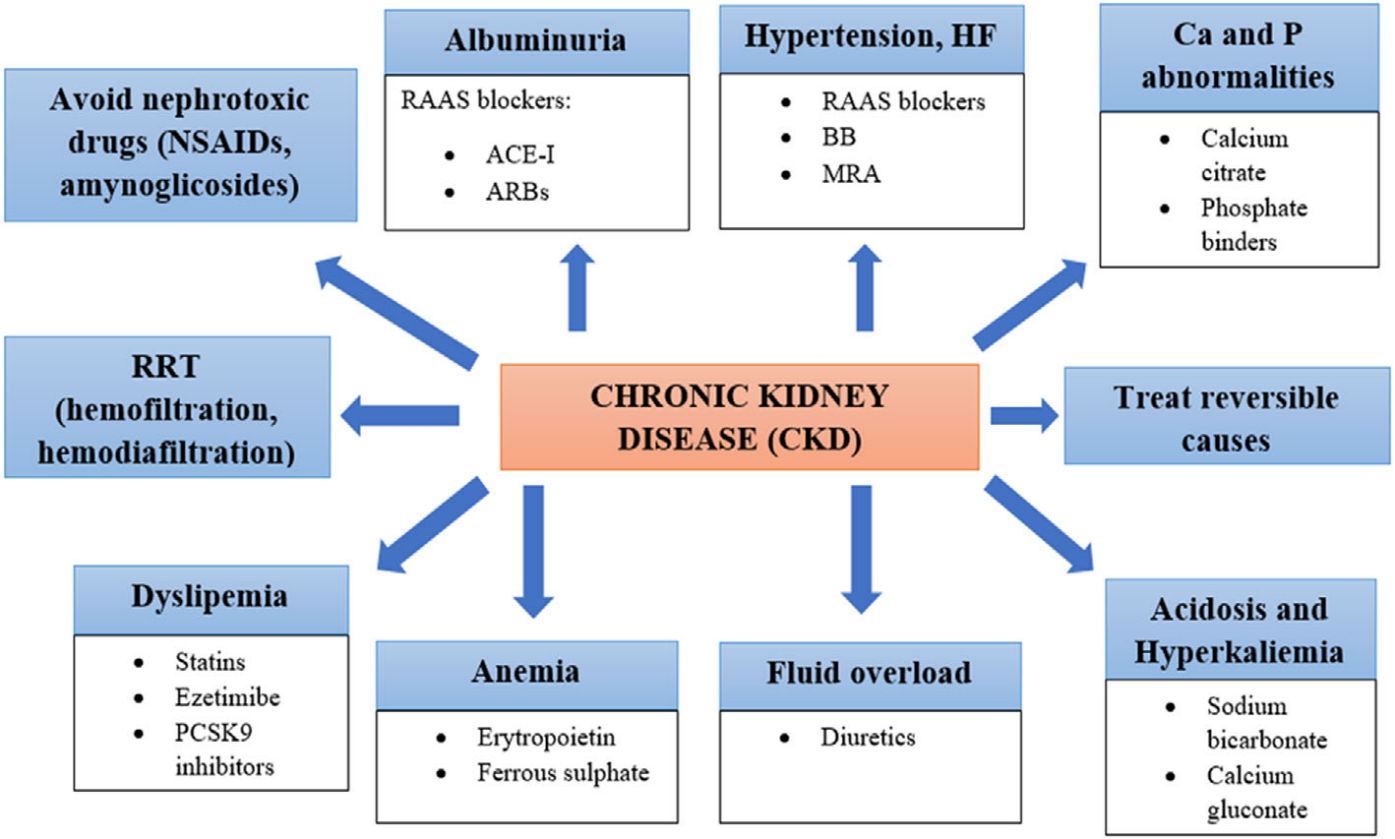
Therapeutic management of CRS‐4. ACE‐I, angiotensin‐converting enzyme inhibitor; ARBs, angiotensin receptor blockers; BB, beta blockers; CKD, chronic kidney disease; MRA, antimineralocorticoid; NSAIDs, non‐steroidal anti‐inflammatory drugs; RAAS, renin–angiotensin–aldosterone system; RRT, renal replacement therapy

**CRS‐4.** Higher sympathetic and RAAS activation are linked to CKD and CRS‐4, which can lead to chronic oxidative stress and inflammation.^65^ ACE‐I proved to enhance CV survival in individuals with mild to moderate kidney dysfunction, regardless of the degree of myocardial impairment.^66^ Their beneficial consequences on ventricular remodeling, neurohormonal activation and hemodynamics might explain why they help dialysis subjects avoid cardiac arrhythmias. Management of ACE‐I, ARBs, and beta‐blockers was strongly linked with better prognosis in ESRD subjects following cardiac arrest by Pun and colleagues.^67^ In a laboratory scenario of CRS in mice, Yang and colleagues found that ARNI therapy significantly protected cardiac and kidney functions, mostly by inhibiting mitochondrial damage, oxidative stress, apoptosis, and fibrosis.^68^ SGLT2 inhibitors reduce blood glucose levels and improve CKD and HF endpoints. SGLT2 inhibitors were found to be effective in subjects with HF or CKD in two recent investigations, EMPEROR‐Reduced^69^ and DAPA‐CKD.^70^ SGLT2 inhibitors have been shown to be effective as an additional treatment for CKD and HF in numerous studies. These drugs may be especially helpful in regulating heart and renal function in people who have both CKD and HF.^71^ The notion of “cardioprotective dialysis” stems from improvements in dialysis equipment that increases hemodynamic stability, minimize inflammatory and oxidative stress, and generate more effective elimination of small and medium toxins. “Hemofiltration” or “hemodiafiltration” procedures have been linked to improve blood pressure control, a reduced incidence of intradialytic hypotension or arrhythmia, improved β2M and phosphorus clearance, lowered oxidative stress and inflammation markers, and reduced hospitalization rate in numerous trials.^72^ In Figure 9 we resume the management of CRS‐4.

### 5.3 Systemic CRS

**FIGURE 10:**
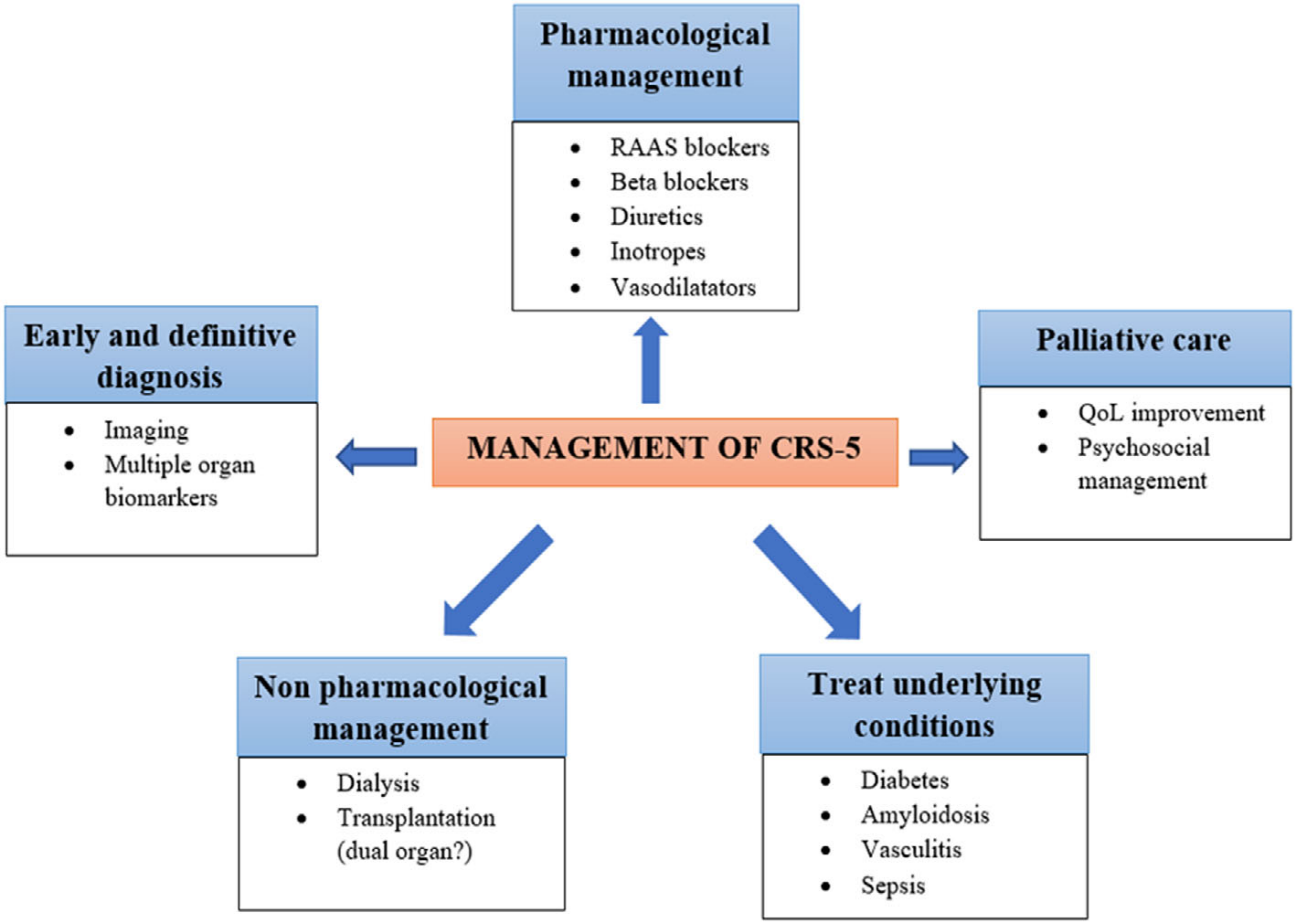
Therapeutic management of CRS‐5. RAAS, renin–angiotensin–aldosterone system; QoL, quality of life

**CRS‐5.** The primary condition administration, as well as the treatment of renal and cardiac renal impairment and its implications, are the target of therapeutic approaches in CRS‐5. In the case of septic CRS‐5, eradication of the cause of infection, antibiotic treatment, and other supportive treatments are recommended.^73^ Early treatment with an intravenous fluid strategy and use of vasopressors or inotropic medications to reverse myocardial depression and systemic vasodilation is critical.^74^ Enhanced CO is a result of higher venous return and better myocardial function that could contribute to a better renal blood perfusion and urine output. RRT is recommended if renal damage continues despite fluid optimization and hemodynamic.^75^ Figure 10 shows the management of CRS‐5.

## 6 CONCLUSIONS

CRS refers to a group of acute and chronic diseases in which the main damaged organ can be both the heart or the kidney. A knowledge of the relationship between the heart and the kidney has therapeutic consequences in both chronic and acute conditions. The level of awareness and complexity of management required to provide the optimal treatment for these individuals necessitates a multidisciplinary strategy centered on pathogenesis. The capacity to recognize and characterize the pathophysiology of CRS will aid to improve the prognosis of these difficult subject to manage.

## CONFLICT OF INTEREST

The authors declare that they have no conflicts of interest.

## Data Availability

Data sharing not applicable to this article as no datasets were generated or analysed during the current study.
